# Association between Blood Pressure and Birth Weight among Rural South African Children: Ellisras Longitudinal Study

**DOI:** 10.3390/ijerph14090974

**Published:** 2017-08-29

**Authors:** Kotsedi Monyeki, Han Kemper, Alfred Mogale, Leon Hay, Machoene Sekgala, Tshephang Mashiane, Suzan Monyeki, Betty Sebati

**Affiliations:** 1 Department of Physiology and Environmental Health, University of Limpopo, Polokwane 0727, South Africa; dsekgala@hsrc.ac.za (M.S.); tshephangmashiane@gmail.com (T.M.); msmonyeki@yahoo.com (S.M.); rbettysebati76@gmail.com (B.S.); 2 VU University Medical Center, Institute for Health and Care Research, 1081 HV Amsterdam, The Netherlands; hancgkemper@upcmail.nl; 3 Department of Biochemistry, Sefako Makgatho Health Science University, Pretoria 0204, South Africa; alfred.mogale@smu.ac.za; 4 Human Physiology Department, Sefako Makgatho Health Science University, Pretoria 0204, South Africa; leon.hay@smu.ac.za; 5 Population Health, Health Systems and Innovation, Human Science Research Council, Pretoria 0083, South Africa

**Keywords:** birth weight, underweight, mid upper arm circumference, blood pressure, rural South African children

## Abstract

The aim of this cross-sectional study was to investigate the association between birth weight, underweight, and blood pressure (BP) among Ellisras rural children aged between 5 and 15 years. Data were collected from 528 respondents who participated in the Ellisras Longitudinal Study (ELS) and had their birth weight recorded on their health clinic card. Standard procedure was used to measure the anthropometric measurements and BP. Linear regression was used to assess BP, underweight variables, and birth weight. Logistic regression was used to assess the association of hypertension risks, low birth weight, and underweight. The association between birth weight and BP was not statistically significant. There was a significant (*p* < 0.05) association between mean BP and the sum of four skinfolds (β = 0.26, 95% CI 0.15–0.23) even after adjusting for age (β = 0.18, 95% CI 0.01–0.22). Hypertension was significantly associated with weight for age z-scores (OR = 5.13, 95% CI 1.89–13.92) even after adjusting for age and sex (OR = 5.26, 95% CI 1.93–14.34). BP was significantly associated with the sum of four skinfolds, but not birth weight. Hypertension was significantly associated with underweight. Longitudinal studies should confirm whether the changes in body weight we found can influence the risk of cardiovascular diseases.

## 1. Introduction

Hypertension is known to be the third major risk factor of deaths among adolescents, and recent studies have shown that the prevalence of hypertension in children is increasing from 5.4% to 19.4% [1,2,3]. Analysis from middle- and low-income countries indicate that about two-thirds of strokes and more than half of ischaemic heart disease (IHD) cases are attributable to the high prevalence of hypertension [4]. This is due to elevated left ventricular mass and thickening of the carotid arterial wall [5]. The determinants of hypertension in childhood and adolescents have been a subject of considerable research attention over the past few decades. Numerous epidemiological studies have suggested that a risk of attaining hypertension and cardiovascular diseases (CVD) later in life can be linked with blood pressure (BP) and birth weight [6,7]. It is commonly known that cardiovascular diseases’ risk factors that result in morbidity and mortality in adulthood begin earlier in life [1,2]. Poor utero growth was also associated with the high prevalence of cardiovascular diseases in adults [8]. Factors affecting BP in childhood may have negative impacts on adult health.

Numerous studies have suggested that birth weight is a significant factor for developing BP in a later stage of life [3,6,9]. It has been reported that a 1-kg increase in birth weight was associated with a 1–2-mmHg decrease in systolic BP among children [10]. To date, the association between birth weight and BP has not been reported in rural South African children. There is convincing evidence that BP commonly increases with body growth in childhood and adolescence, while postnatal weight gain could have more influence on BP than birth weight [11,12,13,14]. Ellisras rural children, however, were reported to be undernourished over time, with a low prevalence of hypertension and overweight [8]. Therefore, this study aimed to (1) assess the risks of hypertension in children who are underweight and those who were LBW (low birth weight), and (2) assess the association between birth weight, underweight, and BP in rural children aged 5 to 15 years.

## 2. Methods

The more detailed Ellisras Longitudinal Study (ELS) design has been published elsewhere [15]. For the current cross-sectional analysis, ELS respondents aged 5 to 15 years participated in the analysis. Birth weights were recorded from immunization cards. Out of 2238 ELS respondents at base line, birth weight was recorded for only 605 children. Some (20%) of ELS children were born at home, while some (10%) of the primary health care services were dysfunctional, resulting in these children’s birth weights not being recorded on their immunization cards. A total of 528 (299 males and 229 females) children, whose birth weight measurements were recorded on their health clinic cards, were included in the analysis, together with the first BP and anthropometric measurements of May 1999.

### 2.1. Anthropometry

The procedure for the International Society for the Advancement of Kinanthropometry was used to measure anthropometric measurements in all children [16]. An electronic scale was used to measure weight to the nearest 0.1 kg, and height was measured using a Martin Anthropometer to the nearest 0.1 cm. Slim Guide skinfold caliper was used to measure skinfolds [16].

Birth weight of 2500 g or less, regardless of gestational age, was regarded as the cut-off point for low birth weight (LBW) [17]. Over fatness was derived as the sum of four skinfold thicknesses above the 90th percentile for age and sex [17]. Malnutrition was assessed by using mid upper arm circumference (MUAC) cut-off points, which were used to indicate underweight in young children [18]. Measures of height-for-age (HAZ), weight-for-age (WAZ), and weight-for-height (WHZ) expressed as Z scores of National Health and Nutrition Examination Survey III were used [19]. Z score values of less than −2 of height-for-age (stunting), weight-for-age (underweight), and weight-for-height (wasting) were used to determine the prevalence of stunting, underweight, and wasting, respectively [20].

### 2.2. Blood Pressure

Electronic Micronta monitoring kit was used to measure three BP readings of systolic blood pressure (SBP) and diastolic pressure (DBP) at an interval of five minutes apart [12]. The bladder of the device contains an electronic infrasonic transducer to monitor the BP and pulse rate on the screen. The Electronic Micronta monitoring kit instrument was designed for research and clinical purposes. Mean BP was defined according to Norton & Olds [16] as:

$$\mathrm{mean}\;\mathrm{BP}=\frac{2}{3}\left(\mathrm{DBP}\right)+\frac{1}{3}\left(\mathrm{SBP}\right)$$

Hypertension was defined as the measurements of SBP and DBP levels greater than or equal to the 95th percentile of height- and sex-adjusted reference levels [12].

### 2.3. Ethical Considerations

This study is a part of ongoing ELS that commenced in 1996 with ethical clearance number MREC/P/204/2013: IR.

### 2.4. Quality Control

The absolute and relative values for intra- and inter-tester technical errors of measurements (% TEM) for stature ranged from 0.04 to 4.16 cm (0.2–5.01%). The reliability and validity of anthropometric and physical fitness measurements were reported elsewhere [15].

### 2.5. Statistical Analysis

Descriptive statistics was provided for birth information, absolute body size, WAZ, HAZ, and WHZ Z-scores and BP for Ellisras rural children aged 5 to 15 years by sex. We assessed the ability of low birth weight (LBW) and nutritional status to differentiate hypertensive children. We then formed sex-specific receiver operating characteristics (ROC) curves and used the conforming area under curves (AUC) to assess the ability of low birth weight and nutritional status to identify children with hypertension. The ROC curve is defined as a plot of true-positive rate (sensitivity) against the false-positive rate (1-specificity). The best test has an ROC skewed at the upper left corner with an AUC of 1, while an AUC of 0.5 shows that the test performs no better than a chance [21,22,23,24]. Linear regression models were used to evaluate the existing associations between mean BP, birth weight, and body composition parameters adjusted for age and sex. Logistic regression analysis was applied in order to determine the risk of hypertension in underweight and low birth weight in children. The Statistical Package for the Social Sciences (SPSS 22.0) (SPSS Inc., Chicago, IL, USA) was used for all statistical analysis, *p* < 0.05.

## 3. Results

**Figure 1:**
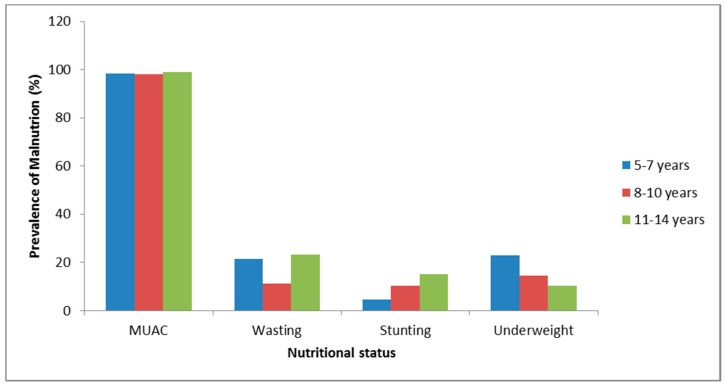
Prevalence of malnutrition (underweight) based on MUAC and Z-scores among Ellisras rural children aged 5–15 years. MUAC: mid upper arm circumference.

Figure 1 shows the prevalence of malnutrition based on MUAC, wasting, stunting, and underweight in different age groups. There was a high prevalence of wasting (21%) and stunting (16%) among the group aged 11–15 years, while there was a high prevalence of underweight (22%) among the group aged 5–7 years.

Table 1 shows descriptive statistics by age groups and sex for birth information, BP, and absolute body size variables of the Ellisras rural children. Birth weight showed no mean significant difference between the age groups. There was a significant difference between males and females in body weight and height-for-age in the group aged 11–15 years, while HAZ showed a significant (*p* < 0.05) difference between males and females in the group aged 5–7 years.

**Figure 2:**
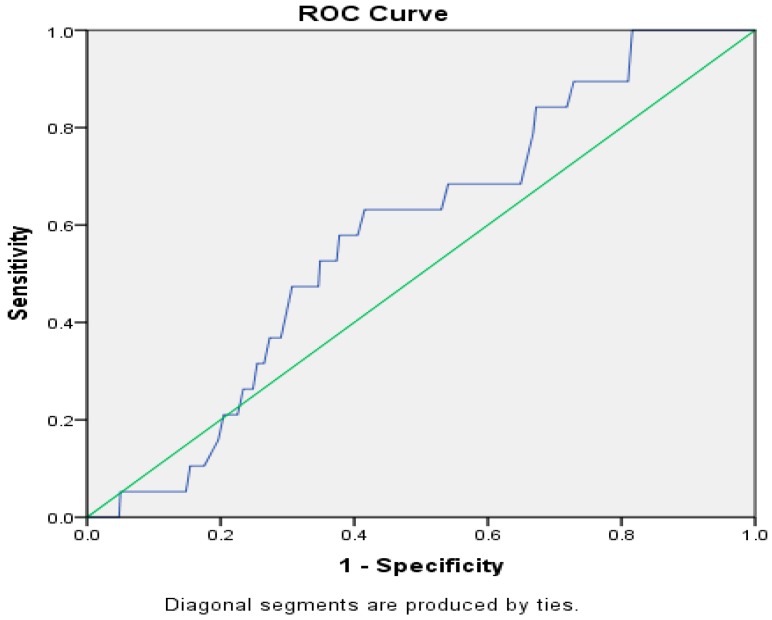
Area Under Curve operating characteristics curves showing the ability of nutritional status and low birth weight to identify Ellisras rural children with hypertension. SEN = Sensitivity, SPE = Specificity, *p* < 0.05.

The receiver operating characteristic (ROC) curve analyses on Figure 2 showed the ability of under nutrition to correctly identify children with high BP. AUC for under nutrition (0.78, 95% CI 0.62–0.94) was statistically significant (*p* < 0.05) for hypertension, while birth weight showed non-significant results for both high BP and over fatness.

Table 2 shows the linear regression model, 95% CI, and *p*-value for the association of mean BP with low birth weight, nutritional status and the sum of four skinfolds for the Ellisras rural children aged 5 to 15 years. There was a significantly positive (*p* < 0.001) association between mean BP and sum4sf (β = 0.26, 95% CI 0.15–0.36 and β = 0.18, 95% CI 0.01–0.22, after adjusting for age and sex). There was a further significantly positive (*p* < 0.001) association between DBP and gestational age (β = 0.11, 95% CI 0.10–0.31) for non-adjusted values.

Table 3 shows a significant (*p* < 0.05) association between high SBP and wasting (OR = 0.22, 95% CI 0.05–0.96 and OR = 0.22, 95% CI 0.05–0.10), while high DBP (OR = 3.20, 95% CI 1.62–6.32 and OR = 3.10, 95% CI 1.56–6.19) and hypertension (OR = 5.13, 95% CI 1.89–13.92 and OR = 5.26, 95% CI 1.93–14.34) were significantly associated with underweight adjusted and unadjusted for age and sex.

## 4. Discussion

The aim of this study was primarily to investigate the association between BP and birth weight of Ellisras rural children aged between 5 and 15 years. This study confirmed the non-significant association between birth weight and hypertension. It was reported that each 1-kg increment of weight increases the odds of high BP defined as a sex, age, and height specific BP > 90th percentiles, although no association was found between BP and birth weight [13]. In this context, a study on birth weight and hypertension by Barker [7] found that any 1-kg increase in birth weight can be linked with approximately a 3-mmHg reduction in SBP. This suggestion led to the fetal origin hypothesis, which states that different forms of cardiovascular disease originate during fetal life. However, BP was significantly associated with the sum of four skinfolds.

Numerous studies have reported contradictory results on the connotation of birth weight and BP ever since a study by Barker first published the developmental origin of adult disease hypothesis two decades ago [7,25,26,27]. Our study also revealed similar conflicting results, showing no association between birth weight and BP, which could be clarified by a high prevalence of under nutrition observed in the current study. This could confirm mild growth retardation, as reported by Valero De Bernabé et al. [27]. Furthermore, a systematic review on fetal origins by Huxley et al. [11] suggested that head circumference was reported to be associated with BP later in life rather than birth weight, which was also contrary to the findings of the present study [25].

There was a significant association between hypertension and underweight. Similar results were reported elsewhere [28,29,30]. An early life underweight could further increase high BP due to the activation of the renin-angiotensin system [31]. In this current study, there was a significant association between BP and the sum of four skinfolds adjusted for age and sex. Similar findings were reported elsewhere [32,33]. Given the homogeneous nature of our sample, it is difficult to compare the magnitude of the association of the present study with other studies. Changes in the sum of skinfolds over time, as seen in longitudinal studies, could be a better predictor of BP [3,34]. In the current study, the association between birth weight and BP was not statistically significant. However, Moselakgomo et al. [35] reported a positive correlation between BP and stature, body mass index, body fat, and the sum of skinfolds, but not birth weight. The health effect of obesity may be influenced by the anatomic distribution of body fat, which better explains the reasons for hormonal imbalances and environmental stress or genetic factors [1,3,36].

Studies of children are subjected to further confounders’ inclusion of prematurity and growth disparity [37], which we have considered in this analysis. The socioeconomic and marital statuses of parents of the participants, however, were not considered in the analysis. It was found that the mother’s marital status during pregnancy influenced the incidence of low birth weight [27]. However, families from the current study were from rural populations which were characterized by the practice of home births. Birth weight was recorded on health clinic cards, though the actual procedure for measuring was not documented, as it could introduce bias in the analysis of the present study. However, this study provided a baseline estimate based on birth weight, given the low available data in this population. Finally, the small sample size of children with low birth weight and lack of other risk factors for hypertension could introduce bias in the interpretation of the results, given the fact that some of these children were from families with illiterate or deprived parents [2,27]. High systolic BP was significantly associated with wasting, both unadjusted and adjusted for age and sex.

## 5. Conclusions

BP was significantly associated with the sum of four skinfolds, but not with birth weight. There was a high prevalence of underweight in the present sample. Hypertension was significantly associated with underweight. Information on physical activity, dietary intake, and lipid profile could shed more light on the health status of Ellisras rural children.

## Figures and Tables

**Table 1:** Descriptive statistics by age group and sex for birth information, blood pressure (BP), and absolute body size variables for the Ellisras rural children aged 5 to 15 years.

Variables	5–7 Years		8–10 Years		11–15 Years	
	Mean (SD)		Mean (SD)		Mean (SD)	
	Males (N = 38)	Females (N = 27)	Males (N = 138)	Females (N = 117)	Males (N = 102)	Females (N = 76)
**Birth Information**						
Length (cm)	49.11 (5.32)	47.94 (3.97)	50.20 (5.78)	50.13 (7.08)	49.56 (5.45)	50.30 (5.58)
Head circumference (cm)	34.40 (3.00)	37.00 (10.62)	34.64 (5.52)	35.21 (4.40)	34.23 (5.85)	34.85 (9.26)
Birth weight (kg)	3.13 (0.44)	3.17 (0.44)	3.18 (0.55)	3.15 (0.61)	3.23 (0.61)	3.15 (0.47)
Gestational age	38.00 (1.39)	37.77 (1.52)	37.42 (3.80)	37.10 (4.17)	37.20 (4.17)	38.08 (1.38)
**Blood Pressure**						
Systolic mmHg	98.37 (10.06)	99.90 (9.35)	99.13 (11.10)	100.97 (11.6)	98.69 (11.06)	99.26 (10.49)
Diastolic mmHg	64.29 (7.85)	66.55 (7.28)	66.02 (8.10)	67.34 (8.27)	66.39 (8.49)	66.11 (9.08)
mean mmHg	75.65 (7.37)	77.67 (6.91)	77.06 (8.88)	78.55 (8.22)	77.16 (8.41)	77.16 (8.70)
**Absolute Body Size**						
Body weight (kg)	20.89 (2.18)	21.07 (3.62)	25.75 (3.63)	25.36 (4.08)	32.08 (5.07) *	34.17 (6.51) *
Height (cm)	122.64 (5.34)	23.24 (6.42)	133.46 (7.11)	33.51 (6.55)	145.08 (6.74) *	148.05 (6.59) *
Triceps (mm)	6.99 (1.32)	6.77 (1.20)	9.12 (3.19)	8.80 (2.63)	6.16 (1.09)	6.13 (1.30)
Biceps (mm)	4.00 (0.00)	4.00 (0.00)	6.17 (1.93)	6.16 (1.82)	3.39 (0.40)	3.38 (0.45)
Suprailiac (mm)	4.21 (0.91)	4.21 (0.77)	5.76 (2.12)	5.93 (4.91)	4.04 (0.96)	4.00 (0.78)
Subscapular (mm)	5.43 (0.84)	5.50 (0.65)	7.18 (2.34)	7.22 (2.15)	5.22 (0.84)	5.22 (0.77)
Sum4sf (mm)	20.63 (2.31)	20.48 (1.94)	28.23 (8.74)	28.12 (9.25)	18.81 (2.50)	18.72 (2.66)
Underweight (WAZ)	−0.84 (0.57)	−0.58 (0.90)	−0.90 (0.49)	−0.93 (0.47)	−1.02 (0.52)	−1.07 (0.62)
Stunting (HAZ)	−0.11 (0.98) *	1.90 (1.18) *	−0.38 (0.95)	−0.29 (0.78)	−0.65 (0.99)	−0.45 (0.90)
Wasting (WHZ)	−1.14 (0.39)	−1.07 (0.85)	−1.05 (0.42)	−1.03 (0.45)	−0.97 (0.35)	−0.99 (0.64)
MUAC	15.78 (1.03)	15.61 (1.43)	16.85 (1.20)	16.90 (1.43)	18.31 (1.69)	19.05 (2.28)

* *p* < 0.05; WAZ = weight-for-age; HAZ = height-for-age; WHZ = weight-for-height; MUAC = mid upper arm circumference; Sum4sf = Sum of four skinfolds.

**Table 2:** Linear regression model, 95% CI, and *p*-value for the association of mean BP, birth information, and the sum of four skinfolds for Ellisras rural children aged 5 to 15 years.

Variables	Mean Blood Pressure					
	Non-Adjusted			Adjusted for Age and Sex		
	β	*p*-Value	95% CI	β	*p*-Value	95% CI
Birth weight (kg)	−0.43	0.44	−1.55–0.69	−0.56	0.29	53.95–63.33
Head circumference (cm)	0.01	0.75	−0.08–0.11	0.04	0.34	−0.05–0.14
Gestational age	−0.02	0.84	−0.23–0.18	−0.02	0.82	−0.23–0.18
Sum4sf (mm)	0.26	0.00	0.15–0.36	0.18	0.03	0.01–0.22
Underweight (WAZ)	−0.01	0.99	−1.38–1.37	0.14	0.83	−1.26–1.55
Stunting (HAZ)	−0.13	0.71	−0.84–0.57	−0.10	0.80	−0.88–0.68
Wasting (WHZ)	−0.94	0.24	−2.49–0.62	−1.05	0.19	−0.27–0.61
MUAC	0.26	0.19	−0.13–0.65	0.11	0.65	−0.37–0.59
Systolic BP						
Birth weight (kg)	0.28	0.74	−1.34–1.94	0.07	0.93	−1.49–1.63
Head circumference (cm)	−0.03	0.70	−0.18–0.12	0.01	0.91	−0.13–0.15
Gestational age	−0.11	0.44	−0.38–0.16	−0.12	0.41	−0.39–0.16
Sum4sf (mm)	0.35	0.00	0.20–0.51	0.18	0.03	0.02–0.34
Underweight (WAZ)	−0.54	0.54	−2.30–1.21	−0.32	0.73	−2.12–1.49
Stunting (HAZ)	−0.18	0.70	−1.08–0.73	−0.12	0.81	−1.11–0.86
Wasting (WHZ)	−1.56	0.13	−3.55–0.44	−1.62	0.11	−3.61–0.38
MUAC	0.25	0.34	−0.27–0.77	0.12	0.72	−0.52–0.76
Diastolic BP						
Birth weight (kg)	−0.79	0.17	−1.90–0.33	−0.87	0.11	−1.93–0.20
Head circumference (cm)	0.04	0.45	−0.06–0.14	0.06	0.19	−0.03–0.16
Gestational age	0.02	0.83	−0.19–0.23	0.02	0.84	−0.19–0.23
Sum4sf (mm)	0.21	0.00	0.10–0.31	0.09	0.12	−0.02–0.19
Underweight (WAZ)	−0.42	0.54	−1.75–0.91	−0.12	0.86	−1.49–1.25
Stunting (HAZ)	0.04	0.91	−0.65–0.73	0.15	0.69	−0.60–0.90
Wasting (WHZ)	−1.24	0.11	−2.76–0.28	−1.29	0.09	−2.81–0.23
MUAC	0.26	0.19	−0.13–0.66	0.11	0.67	−0.38–0.74

WAZ = weight-for-age; HAZ = height-for-age; WHZ = weight-for-height; MUAC = mid upper arm circumference; Sum4sf = sum of four skinfolds.

**Table 3:** Logistic regression model for non-adjusted and adjustable regression coefficients (β), 95%CIs, and *p*-values associating hypertension with low birth weight, and nutritional status (WHZ, HAZ, WAZ).

Variables	Unadjusted			Adjusted for Age and Sex		
	OR	*p*-Value	95% CI	OR	*p*-Value	95% CI
**High Systolic BP**						
Low birth weight	1.34	0.516	0.56–3.22	1.31	0.553	0.54–3.16
Wasting (WHZ)	0.22	0.044	0.05–0.96	0.22	0.048	0.05–0.10
Stunting (HAZ)	0.74	0.633	0.22–2.54	0.85	0.800	0.24–2.10
Underweight (WAZ)	2.23	0.068	0.94–5.27	2.21	0.074	0.93–5.27
**High Diastolic BP**						
Low birth weight	1.03	0.949	0.46–2.31	0.96	0.913	0.42–2.17
Wasting	0.69	0.362	0.31–1.53	0.66	0.326	0.29–1.50
Stunting	1.57	0.289	0.68–3.59	1.78	0.188	0.76–4.18
Underweight	3.20	0.001	1.62–6.32	3.10	0.001	1.56–6.19
**Hypertension**						
Low birth weight	1.07	0.914	0.30–3.90	1.03	0.968	0.28–3.78
Wasting (WHZ)	0.00	0.997	-	0.00	0.996	-
Stunting (HAZ)	0.56	0.577	0.07–4.37	0.63	0.665	0.08–5.05
Underweight (WAZ)	5.13	0.001	1.89–13.92	5.26	0.001	1.93–14.34

OR = odds ratio, CI = confidence interval, BP = blood pressure.
